# Dr. Eugene Orville Bardwell

**Published:** 1911-04

**Authors:** 


Dr. Eugene Orville Bardwell, died at his home in Emporium. Pa., January 4, 1911, from pneumonia, aged 55 years. He graduated from the University of Buffalo in 1879.

